# LiverScreen project: study protocol for screening for liver fibrosis in the general population in European countries

**DOI:** 10.1186/s12889-022-13724-6

**Published:** 2022-07-19

**Authors:** Isabel Graupera, Maja Thiele, Ann T. Ma, Miquel Serra-Burriel, Judit Pich, Núria Fabrellas, Llorenç Caballeria, Robert J. de Knegt, Ivica Grgurevic, Mathias Reichert, Dominique Roulot, Jörn M. Schattenberg, Juan M. Pericas, Paolo Angeli, Emmanuel A. Tsochatzis, Indra Neil Guha, Montserrat Garcia-Retortillo, Rosa M. Morillas, Rosario Hernández, Jordi Hoyo, Matilde Fuentes, Anita Madir, Adrià Juanola, Anna Soria, Marta Juan, Marta Carol, Alba Diaz, Sönke Detlefsen, Pere Toran, Guillem Pera, Céline Fournier, Anne Llorca, Phillip N. Newsome, Michael Manns, Harry J. de Koning, Feliu Serra-Burriel, Fernando Cucchietti, Anita Arslanow, Marko Korenjak, Laurens van Kleef, Josep Lluis Falcó, Patrick S. Kamath, Tom H. Karlsen, Laurent Castera, Frank Lammert, Aleksander Krag, Pere Ginès, for the LiverScreen Consortium investigators, Marifé Alvarez, Peter Andersen, Paolo Angeli, Alba Ardèvol, Anita Arslanow, Luca Beggiato, Zahia Ben Abdesselam, Lucy Bennett, Bajiha Boutouria, Alessandra Brocca, M. Teresa Broquetas, Llorenc Caballeria, Valeria Calvino, Judith Camacho, Aura Capdevila, Marta Carol, Laurent Castera, Marta Cervera, Fernando Cucchietti, Anna de Fuentes, Rob de Knegt, Sonke Detlefsen, Alba Diaz, José Diéguez Bande, Vanessa Esnault, Núria Fabrellas, Josep lluis Falco, Rosa Fernández, Celine Fournier, Matilde Fuentes, Peter Galle, Edgar García, Montserrat García-Retortillo, Esther Garrido, Pere Ginès, Rosa Gordillo Medina, Jordi Gratacós-Gines, Isabel Graupera, Ivica Grgurevic, Indra Neil Guha, Eva Guix, Rebecca Harris, Elena Hernández Boluda, Rosario Hernández-Ibañez, Jordi Hoyo, Arfan Ikram, Simone Incicco, Mads Israelsen, Marta Juan, Adria Juanola, Ralf Kaiser, Patrick S. Kamath, Tom H. Karlsen, Maria Kjærgaard, Harry J. de Koning, Marko Korenjak, Aleksander Krag, Johanne Kragh Hansen, Marcin Krawczyk, Irina Lambert, Frank Lammert, Philippe Laboulaye, Simon Langkjær Sørensen, Cristina Laserna-Jiménez, Sonia Lazaro Pi, Elsa Ledain, Vincent Levy, Vanessa Londoño, Guirec Loyer, Anne Llorca, Ann T. Ma, Anita Madir, Michael Manns, Denise Marshall, M. Lluïsa Martí, Sara Martínez, Ricard Martínez Sala, Roser Masa Font, Jane Møller Jensen, Rosa M. Morillas, Laura Muñoz, Ruth Nadal, Laura Napoleone, J. M. Navarrete, Phillip N. Newsome, Vibeke Nielsen, Martina Pérez, Juan Manuel Pericas Pulido, Salvatore Piano, Judit Pich, Judit Presas Escobet, Elisa Pose, Katrine Prier Lindvig, Matthias Reichert, Carlota Riba, Dominique Roulot, Ana Belén Rubio, Maria Sánchez-Morata, Jörn Schattenberg, Feliu Serra-Burriel, Miquel Serra-Burriel, Louise Skovborg Just, Milan Sonneveld, Anna Soria, Christiane Stern, Patricia Such, Maja Thiele, Pere Toran, Antoni Torrejón, Marta Tonon, Emmanuel A. Tsochatzis, Laurens van Kleef, Paulien van Wijngaarden, Vanessa Velázquez, Ana Viu, Susanne Nicole Weber, Tracey Wildsmith

**Affiliations:** 1 Liver Unit Hospital Clínic, University of Barcelona, Barcelona, Catalonia Spain; 2 Institut D’Investigacions Biomèdiques August Pi I Sunyer (IDIBAPS), Barcelona, Spain; 3 Centro de Investigación En Red de Enfermedades Hepáticas Y Digestivas (Ciberehd), Barcelona, Spain; 4 Faculty of Medicine and Health Sciences, University of Barcelona, Barcelona, Spain; 5 Centre for Liver Research, Department of Gastroenterology and Hepatology, Odense University Hospital, and Institute for Clinical Research, University of Southern Denmark Odense, Odense, Denmark; 6 Epidemiology, Statistics, and Prevention Institute, University of Zurich, Zurich, Switzerland; 7 Clinical Trial Unit, Hospital Clínic, 08036 Barcelona, Spain; 8 Unitat de Suport a la Recerca Metropolitana Nord, Fundació Institut Universitari per a la recerca a l’Atenció Primària de Salut Jordi Gol i Gurina (IDIAPJGol), Metropolitana Nord, IDIAP Jordi Gol, ICS Institut Català de la Salut, Barcelona, Spain; 9 Department of Gastroenterology and Hepatology, Erasmus MC University Medical Centre, Rotterdam, the Netherlands; 10 Department of Gastroenterology, Hepatology and Clinical Nutrition, University Hospital Dubrava, University of Zagreb School of Medicine and Faculty of Pharmacy and Biochemistry, Zagreb, Croatia; 11 Department of Medicine II, Saarland University Medical Center, Homburg, Germany; 12 Unité d’Hépatologie, Hôpital Avicenne, AP-HP, Université Paris 13, Bobigny, France; 13 Metabolic Liver Research Program, Department of Internal Medicine I, University Medical Centre of the Johannes Gutenberg, Mainz, Germany; 14 Liver Unit, Department of Internal Medicine, Hospital Universitari Vall d´Hebron, Vall d’Hebron Institut de Recerca (VHIR) , Vall d’Hebron Barcelona Hospital Campus, Barcelona, Spain; 15 Universitat Autònoma de Barcelona, Barcelona, Spain; 16 Unit of Internal Medicine and Hepatology (UIMH), Department of Medicine (DIMED), University-Teaching Hospital of Padova, Padua, Italy; 17 UCL Institute for Liver and Digestive Health, Royal Free Hospital, University College of London (UCL), London, UK; 18 NIHR Nottingham Biomedical Research University Mainz Centre, Nottingham University Hospitals NHS Trust and the University of Nottingham, Nottingham, UK; 19 Liver Section, Gastroenterology Department, Hospital del Mar, Department of Medicine, IMIM, Barcelona, Spain; 20 Liver Unit, Hospital Germans Trias i Pujol, IGTP, Badalona, Spain; 21 Institut Catala de la Salut (ICS). BCN. Ambit d’Atencio Primaria, Barcelona, Spain; 22 Department of Pathology. Centre of Biomedical Diagnosis. Hospital Cínic, Barcelona, Spain; 23 Department of Pathology, Odense University Hospital (OUH), University of Southern Denmark, Odense, Denmark; 24 Echosens, Paris, France; 25 National Institute for Health Research Biomedical Research Centre at University Hospitals Birmingham NHS Foundation Trust and the University of Birmingham, Birmingham, UK; 26 Health Sciences, Hannover Medical School MHH, Hannover, Germany; 27 Department of Public Health, Erasmus University Medical Center, Rotterdam, the Netherlands; 28 Barcelona Super Computing Center (BSC), Barcelona, Spain; 29 European Liver Patients’ Association, Brussels, Belgium; 30 Genesis Biomed, Barcelona, Spain; 31 Division of Gastroenterology and Hepatology, Mayo Clinic College of Medicine and Science, Rochester, Minnesota USA; 32 Oslo University hospital, Oslo, Norway; 33 Department of Hepatology, Hôpital Beaujon, Assistance Publique-Hôpitaux de Paris, Clichy, Université de Paris, Paris, France; 34 Institute for Occupational Medicine and Public Health, Saarland University, Homburg, Germany; 35 Hannover Medical School (MHH), Hannover, Germany

**Keywords:** Cirrhosis, Screening, Liver fibrosis, Chronic liver disease, NAFLD, NASH, Vibration-controlled transient elastography

## Abstract

**Background:**

The development of liver cirrhosis is usually an asymptomatic process until late stages when complications occur. The potential reversibility of the disease is dependent on early diagnosis of liver fibrosis and timely targeted treatment. Recently, the use of non-invasive tools has been suggested for screening of liver fibrosis, especially in subjects with risk factors for chronic liver disease. Nevertheless, large population-based studies with cost-effectiveness analyses are still lacking to support the widespread use of such tools. The aim of this study is to investigate whether non-invasive liver stiffness measurement in the general population is useful to identify subjects with asymptomatic, advanced chronic liver disease.

**Methods:**

This study aims to include 30,000 subjects from eight European countries. Subjects from the general population aged ≥ 40 years without known liver disease will be invited to participate in the study either through phone calls/letters or through their primary care center. In the first study visit, subjects will undergo bloodwork as well as hepatic fat quantification and liver stiffness measurement (LSM) by vibration-controlled transient elastography. If LSM is ≥ 8 kPa and/or if ALT levels are ≥1.5 x upper limit of normal, subjects will be referred to hospital for further evaluation and consideration of liver biopsy. The primary outcome is the percentage of subjects with LSM ≥ 8kPa. In addition, a health economic evaluation will be performed to assess the cost-effectiveness and budget impact of such an intervention. The project is funded by the European Commission H2020 program.

**Discussion:**

This study comes at an especially important time, as the burden of chronic liver diseases is expected to increase in the coming years. There is consequently an urgent need to change our current approach, from diagnosing the disease late when the impact of interventions may be limited to diagnosing the disease earlier, when the patient is asymptomatic and free of complications, and the disease potentially reversible. Ultimately, the LiverScreen study will serve as a basis from which diagnostic pathways can be developed and adapted to the specific socio-economic and healthcare conditions in each country.

**Trial registration:**

This study is registered on Clinicaltrials.gov (NCT03789825).

**Supplementary Information:**

The online version contains supplementary material available at 10.1186/s12889-022-13724-6.

## Background

Liver cirrhosis is the end stage of chronic liver diseases and is a major cause of morbidity and mortality worldwide. According to data from the Global Burden of Disease Study, cirrhosis is the 11^th^ cause of death globally and the 7^th^ and 12^th^ leading cause of disability-associated life years (DALY) in people aged 50-74 years and 25-49 years respectively [1]. It is also the second cause of years of working life lost in Europe [2]. The main etiologies of cirrhosis are hepatitis B and C virus infection, increased alcohol consumption and non-alcoholic fatty liver disease (NAFLD), the latter of which is often associated with obesity and type 2 diabetes mellitus [3].

The development of cirrhosis, regardless of its cause, usually occurs very slowly over 2-3 decades, a period during which collagen relentlessly deposits within the liver until the normal liver architecture is disrupted and portal hypertension develops. In general, patients are not diagnosed during this period because fibrosis deposition is an asymptomatic process [3]. As such, most cases of cirrhosis are diagnosed when patients develop complications related to portal hypertension, liver failure or liver cancer. No global strategy exists for the early detection of cirrhosis before such decompensation or cancer occurs. Nevertheless, the main factor predicting long-term outcomes in patients with chronic liver disease is the existence of liver fibrosis [4, 5], which highlights the importance of identifying the disease early. Although some studies have demonstrated the potential reversibility of liver fibrosis after treating the etiology, the actual effectiveness of treatments decrease in late stages compared to early stages of fibrosis [6]. Emphasis should therefore be put on early diagnosis of disease to maximize its potential reversibility.

Standard liver biochemical tests such as serum aminotransferases, or liver ultrasound, are not accurate methods to detect fibrosis [3]. In recent years, several non-invasive methods assessing the presence and severity of liver fibrosis have been developed. These methods rely either on a blood test or on liver stiffness measurement (LSM), using vibration-controlled transient elastography (VCTE) (FibroScan®, Echosens, Paris), which is the most used and validated non-invasive tool for staging chronic liver diseases [7–10]. VCTE is a point-of-care technique that can be performed by nurses after a short training period, and consequently a large number of people can be assessed [11]. While VCTE is available in many liver centers, it is mostly not available in primary care settings where the early detection of liver fibrosis should in fact take place. This technique is particularly suited for the early detection of chronic liver disease either in the general or in high-risk populations, particularly in patients with obesity, diabetes or increased alcohol consumption [9]. Moreover, previous data suggest that the strategy of liver fibrosis screening using TE may be cost-effective [12] but require further validation.

### Study Rationale and Hypothesis

Different recommendations have been made by the scientific community to assess liver fibrosis, especially in at-risk populations, using non-invasive tools [10, 13, 14]. However, these recommendations are based on previous studies that either had insufficient sample size, did not systematically confirm the diagnosis with a liver biopsy, or were not specifically designed to assess the cost-effective strategy in terms of health outcomes and treatment costs in either general or at-risk population [15]. Therefore, we hypothesize that a screening program using a non-invasive method for the diagnosis of liver fibrosis may be useful in detecting asymptomatic subjects with advanced liver fibrosis. This early detection of disease would allow the implementation of treatment with the aim of halting progression or even favoring regression of fibrosis, thus preventing the development of hard clinical outcomes related to the liver disease, such as liver cirrhosis, liver cancer, or liver-related death.

### Study Aim

This is a population-based study aimed at investigating whether liver stiffness measurement using transient elastography is useful to identify subjects with asymptomatic significant chronic liver disease in the general population.

### Study design

This study is a single group screening study. It has been registered retrospectively on Clinicaltrials.gov (NCT03789825) the 14^th^ of November 2018. First patient included was in May 2018.

**Fig. 1:**
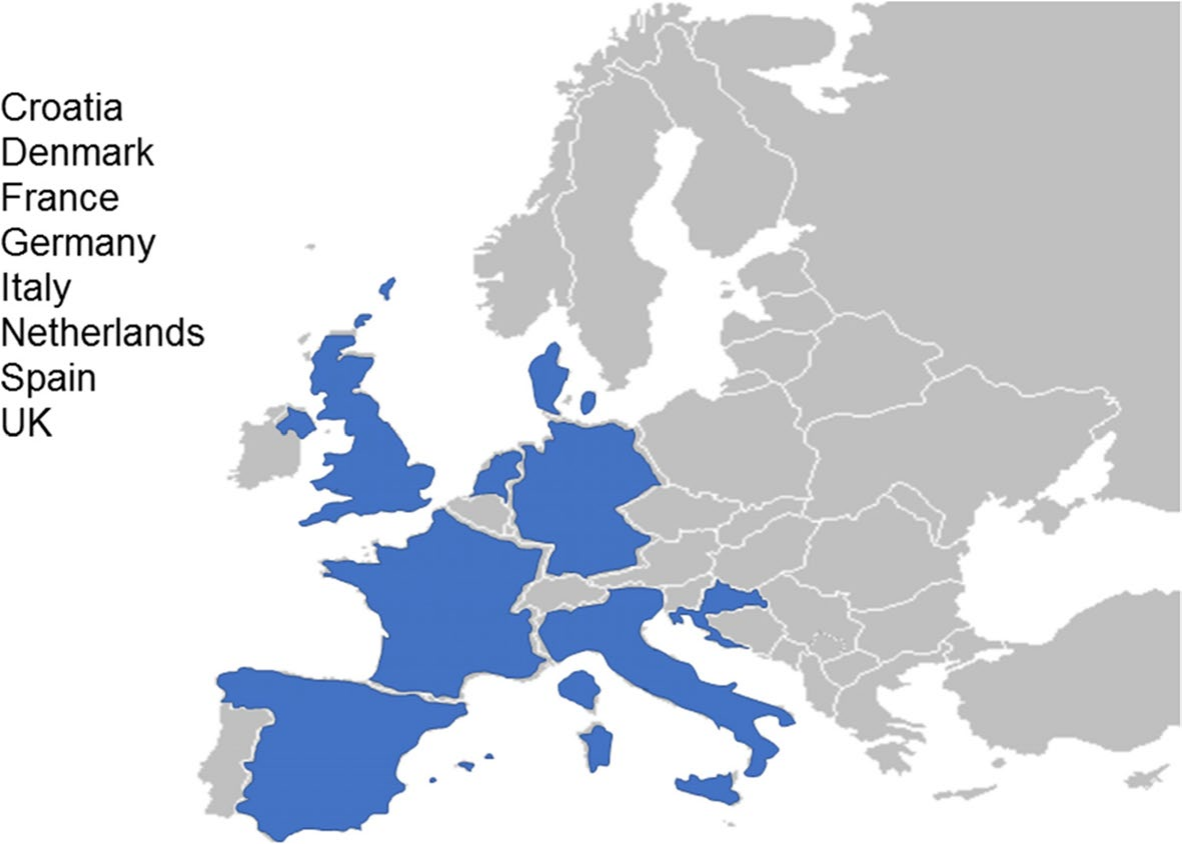
Countries included in the LiverScreen project

**Fig. 2:**
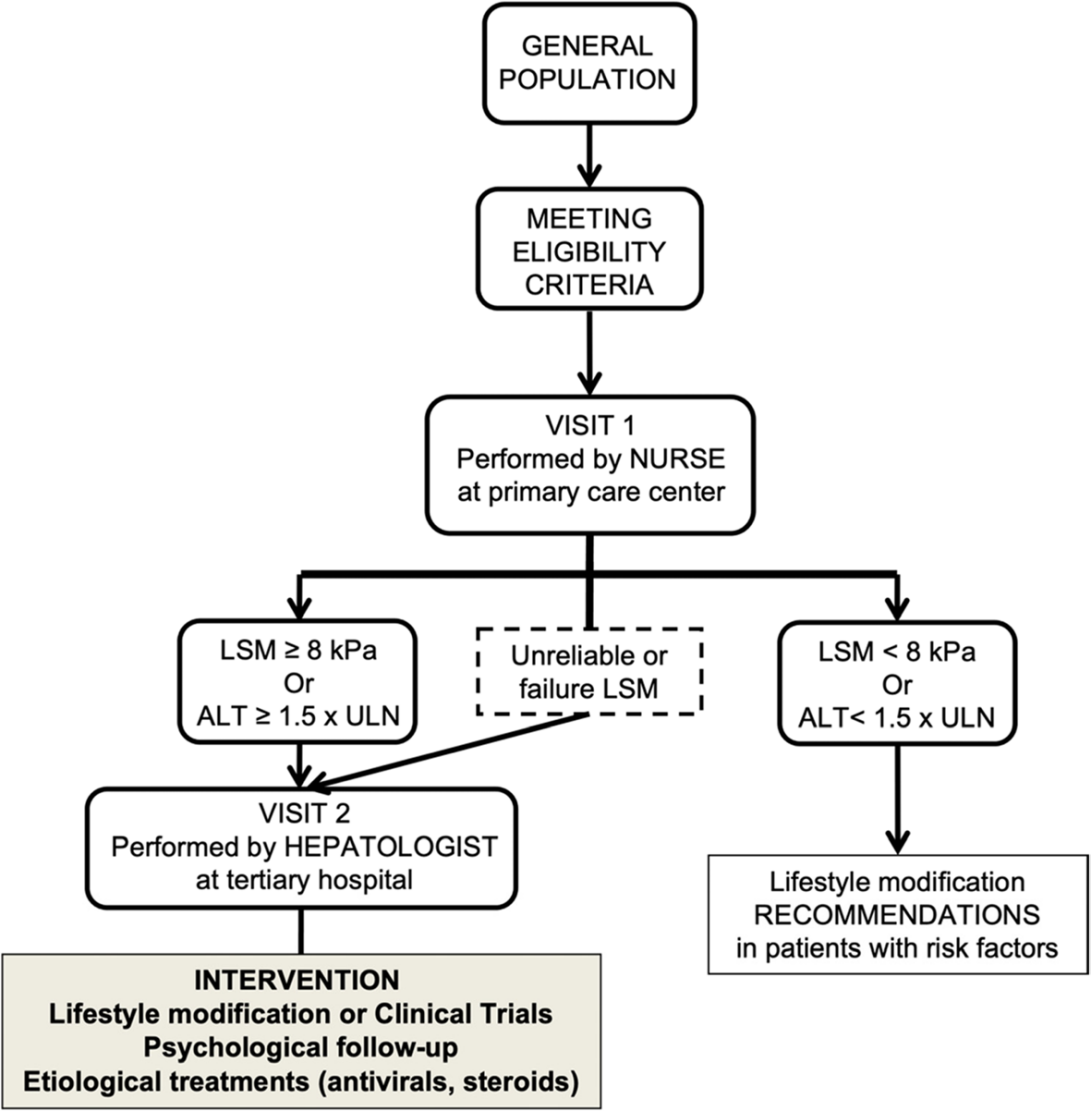
Study flowchart. LSM, liver stiffness measurement; ULN, upper limit of normal

The present population-based study will include 30,000 subjects in eight European countries (Fig. 1). Eligible participants will be invited to attend at their primary care centre or at a research facility where a nurse will perform visit 1. This visit will include a detailed interview, physical examination, questionnaires related to alcohol consumption, quality of life and health status assessment, venipuncture for liver biochemical tests assessment and biobanking, and VCTE to assess LSM and controlled attenuation parameter (CAP). If participants show a LSM ≥ 8 kPa, unreliable or failed VCTE measurement [16] and/or increased ALT levels (ALT ≥ 1.5 x upper limit of normal value), they will be referred to second visit in a Liver Unit at the academic center for further evaluation (visit 2). Otherwise, the study finishes after completion of visit 1 (Fig. 2). Visit 2 will be completed by hepatologists within the 3 months following visit 1, according to a standardized work-up for liver disease diagnosis and evaluation. Visit 2 will include evaluation of the participant’s medical history, physical examination with complete liver tests, FibroScan examination and abdominal ultrasound. The FibroScan examination at visit 2 will be performed with both probes (M and XL). Afterwards, a liver biopsy will be offered to the patient for diagnosis and staging of liver disease when LSM is above 8 kPa or if chronic liver disease is suspected, as per standard of care.

Following diagnosis and staging at the corresponding university hospital, all patients will be offered standard of care treatment and follow-up. Patients with NAFLD will be offered a lifestyle modification program consisting of diet and exercise targeting weight loss of 10% and controlling risk factors of liver disease progression. Each center will offer the specific program according to its own protocol. Patients with NAFLD participating in the present study can still be included in other studies related to NAFLD that evaluate pharmacological treatments. Such treatment received in the context of clinical trial or other pharmacological interventions will be recorded. Patients with high alcohol consumption will be offered psychological assessment and counseling to stop drinking or, at least, reduce harmful alcohol use. Finally, patients diagnosed with chronic liver disease from other etiologies (autoimmune disease, viral hepatitis, Wilson disease, etc.), will be treated according to guidelines [13, 17–20]. All participants with LSM by VCTE < 8 kPa and ALT levels <1.5 ULN who present risk factors for chronic liver diseases such as increased alcohol consumption or obesity will be counseled on lifestyle modifications by their primary care physician for management of such risk factors.

Participants that fail to attend to visit 2 will be contacted by their primary care physician or nurse and referred again to the second visit. Participants will be asked to consent to be contacted in the future for a re-evaluation at 5 and 10 years after visit 1. Information regarding liver-related clinical outcomes (presence of cirrhosis, decompensated cirrhosis, liver cancer and liver-related death), cardiovascular events, and all causes of death will be collected. Patient assessment will be performed through clinical records (via health care registries or health insurance systems) or telephone contact. No further visits will be performed. These clinical outcomes will be used to inform the cost-effectiveness analysis of the liver fibrosis screening program.

#### Primary outcome measure

Percentage of subjects with LSM by VCTE ≥ 8 kPa at any visit, either with the M or XL probe, in the general population.

#### Secondary outcome measures

1. Percentage of subjects with LSM by VCTE ≥ 8 kPa in the subgroup of patients with risk factors for chronic liver diseases at visit 1 and/or 2.
2. Comparison of liver fibrosis diagnosis accuracy between VCTE and fibrosis scores (including NAFLD fibrosis score, FIB-4, Forns index and APRI score) in the general population and in the subgroup of patients with risk factors for chronic liver disease, at visit 1 and 2.
3. Comparison of liver fibrosis diagnosis accuracy between VCTE, fibrosis scores and liver biopsy in the general population and in the subgroup of patients with risk factors for chronic liver diseases, in patients with a liver biopsy available at visit 2.
4. Percentage of subjects with controlled attenuation parameter (CAP) ≥ 250 dB/m, either with M or XL probe, in the general population and in the subgroup of patients with risk factors for chronic liver diseases at visit 1 and/or 2.
5. Comparison of liver steatosis diagnosis accuracy between CAP and steatosis scores (including fatty liver index (FLI), hepatic steatosis index (HSI), lipid accumulation product (LAP), index of NASH (ION) and NAFLD-liver fat score (NAFLD-LFS)) in the general population and in the subgroup of patients with risk factors for chronic liver diseases at visit 1 and 2.
6. Comparison of liver steatosis diagnosis accuracy between CAP, steatosis scores (including FLI, HSI, LAP, ION and NAFLD-LFS) and liver biopsy in the general population and in the subgroup of patients with risk factors for chronic liver diseases, in patients with liver biopsy available at visit 2.
7. Comparison of liver steatosis diagnosis accuracy between CAP and abdominal ultrasound in the general population and in the subgroup of patients with risk factors for chronic liver diseases, in patients with abdominal ultrasound available at visit 2.
8. Comparison of values obtained with M and XL probes in the assessment of LSM and CAP using VCTE at visit 2.
9. Cost-effectiveness and budgetary impact of a liver fibrosis screening program for liver fibrosis detection in the general population and in the subpopulation with risk factors for chronic liver diseases. Direct and indirect cost savings of early detection of liver fibrosis in subjects with risk factors for chronic liver diseases.
10. Percentage of measurement failure during VCTE examination at visit 1 and 2.
11. Percentage of patients with procedure-related adverse events (adverse events related to VCTE and/or liver biopsy) and serious adverse events during the duration of the study.
12. Detection of common genetic variants, single nucleotide polymorphisms (SNPs), in patients with and without significant hepatic fibrosis or chronic liver diseases, including genome-wide association studies (GWAS) and whole-genome sequencing (WGS) in blood samples.
13. Incidence of cirrhosis, liver cancer, and liver-related death and all-cause of mortality in patients with significant fibrosis and/or severe steatosis after 5 and 10 years from the first evaluation.
14. Incidence of cardiovascular events in patients with significant fibrosis and/or severe steatosis after 5 and 10 years from the first evaluation.

#### Recruitment and enrollment of participants

This is a European multicentre clinical research study undertaken in the general population without known liver disease. Participating countries are Denmark, United Kingdom, France, Germany, Netherlands, Italy, Croatia and Spain. LiverScreen recruiting sites (primary care centres and university hospitals) are described in Additional File 1.

Subjects aged 40 years and older, without known liver disease, from the general population will be eligible for the study. Eligible patients will be identified following EU general data protection regulation (GDPR-2018) and will be contacted either by: 1/ general practitioners or primary care nurses; 2/ electronic records at primary care centres, or; 3/ zip code registry. In the first scenario, general practitioners and primary care nurses will randomly invite walk-in subjects of their respective primary care centres to participate in the study. In the second and third scenarios, subjects will be randomly invited to participate through phone calls or postal letters. In the third scenario, subjects will be electronically invited to participate based on random drawing of social security numbers. Once subjects accept to participate in the study, they will be given an appointment at the primary care center or research facility where they will be informed about the details of the study and enrolled if they meet the inclusion/exclusion criteria outlined below.

##### Inclusion criteria

1. Age ≥ 40 years.
2. Able to give informed consent.

##### Exclusion criteria

Patients meeting 1 or more of the following criteria cannot be selected:

1. Previously known chronic liver disease (including cholestasis). Patients with already known liver steatosis but no diagnosis of liver fibrosis or cirrhosis can be included.
2. Subjects with mental incapacity, language barrier, insufficient social support or any other reason considered by the investigator precluding adequate understanding or cooperation in the study.
3. Subjects with a history of current malignancy including solid tumors and hematologic disorders.
4. Subjects with significant extrahepatic disease that may impair short-term prognosis (including congestive heart failure New York Heart Association Grade IV, chronic obstructive pulmonary disease (COPD) GOLD > 3).
5. Subjects with kidney disease (serum creatinine > 3 mg/dL or undergoing renal replacement therapy).

#### Premature withdrawal

A subject is free to withdraw from the study at any time. In addition, the investigator may decide, for reasons of medical prudence, to remove the subject from the study. The date and reasons for stopping the study will be clearly stated on the subject’s case report form and source document.

#### Measurements and investigation procedures

Measurements and investigations will follow the study plan outlined in Table 1.

**Table 1:** Study procedures

STUDY PROCEDURES	VISIT 1	VISIT 2
Informed consent	**X**	
Inclusion/exclusion criteria	**X**	
Medical/surgical history	**X**	**X**
Vital signs	**X**	**X**
Physical examination	**X**	**X**
Concomitant medication	**X**	**X**
Alcohol consumption questionnaire (AUDIT)	**X**	**X**
Quality of life questionnaires (EuroQoL 5d-3)	**X**	
Health survey (SF-12)	**X**	
**LABORATORY ASSESSMENTS**		
HCV/HBV serologies	**X**	
ALT/AST levels	**X**	**X**
GGT/AP levels	**X**	**X**
Haematology	**X**	
Biochemistry	**X**	
Coagulation	**X**	
Leucocyte and CRP	**X**	
Antibodies for autoimmune and celiac diseases		**X**
Biochemistry: TSH, ceruloplasmin, α1AT, bilirubin		**X**
**BIOBANK**		
Blood samples: plasma, serum, DNA.	**X**	
**VC TRANSIENT ELASTOGRAPHY**	**X**	**X**
**ABDOMINAL ULTRASOUND**		**X**
**LIVER BIOPSY**		**X**
**AE assessment**	**X**	**X**

*AE* adverse event, *AP* alkaline phosphatase, *α1AT* α1-antitrypsin, *CRP* C-reactive protein, *HBV* hepatitis B virus, *HCV* hepatitis C virus, *TSH* thyroid stimulating hormone

**Liver fibrosis** will be estimated by three different methods: LSM, liver fibrosis scores and liver biopsy.

- LSM will be performed in all patients using VCTE. LSM will be performed with M or XL probe following the Automatic Probe Selection tool available on the Fibroscan®. LSM will be repeated at visit 2 in those patients referred to the university hospital. Significant liver fibrosis by VCTE will be defined by a LSM ≥ 8.0 kPa with either M or XL probe.
- Fibrosis scores/serum biomarkers: Four different established liver fibrosis scores using different blood tests will be determined at visit 1: NAFLD fibrosis score (NFS), Forns Index, Fibrosis-4 (FIB-4) and AST to Platelet Ratio Index (APRI). Significant liver fibrosis will be defined when the values of the different scores are above the following cut-offs, according to literature [8, 10]: NFS ≥ 0.676, Forns index ≥ 6.9, FIB-4 ≥ 2.67, APRI ≥ 1.50.
- Liver biopsy. In patients with a liver biopsy available, liver fibrosis will be assessed and defined according to SAF/NASH CRN Score [21] and analyzed by two central readers. Discrepancies between two readers will be re-assessed and discussed until consensus.

**Liver steatosis** will be estimated using four different methods, CAP, abdominal ultrasound, liver steatosis scores and liver biopsy.

- Liver steatosis measured by transient elastography. CAP using VCTE with either M or XL probe will be measured at the time of LSM. Liver steatosis will be diagnosed when the value of CAP ≥ 250 dB/m [22].
- Liver steatosis measured by abdominal ultrasound. Patients referred to visit 2 will be evaluated by the hepatologist, and an abdominal ultrasound will be performed. Liver steatosis assessed by ultrasound will be graded semiquantitatively (mild, moderate and severe) [23].
- Liver steatosis measured by steatosis scores. Five different liver steatosis scores using different blood tests will be determined, including FLI, HSI, LAP, ION, NAFLD- LFS, and compared to CAP values. Significant liver steatosis will be defined when the values of the different scores are above the following cut-offs, according to literature [24]: FLI ≥ 60, HSI > 36, LAP ≥ 80, ION ≥ 22, NAFLD-LFS ≥ -0.640.
- Liver steatosis measured by liver biopsy. In patients with a liver biopsy available, liver steatosis will be assessed in histological samples and defined according to SAF/NASH CRN Score [21] and analyzed by two central readers.

#### Sample size calculation

The sample size for the LiverScreen project has been computed to answer the questions of 1/ the usefulness of TE as a screening method to detect significant liver fibrosis among the European general population ≥ 40 years, and 2/ the differences (if any) in the 5-year incidence of liver clinical outcomes (or cardiovascular events or mortality) between those with and without significant liver fibrosis (LSM ≥ 8 kPa). Because the gold standard for the staging of liver fibrosis remains liver biopsy, the sample size calculation of the study considers the final number of liver biopsies needed to evaluate the concordance between liver biopsy and VCTE. For an estimated prevalence of LSM ≥ 8 kPa of around 3%, assuming a 5% of bilateral alpha error and a statistical power of 95%, a sample size of 28,887 subjects would be required. If 3% of the study population have altered VCTE and only 50% of them agree to have a liver biopsy performed, we will have around 450-500 available liver biopsies. Considering all these factors, the sample size of the study will be of 30,000 subjects.

### Statistical analyses

Categorical parameters will be presented by means of frequencies (%). Continuous parameters will be summarized by mean ± standard deviation or median and interquartile range, where appropriate. All patients having a VCTE examination with valid measurements [8] will be included in the analysis population. Subjects excluded from the analysis will be compared to those having a VCTE examination with valid measurements using the Chi squared test and t-test for categorical or continuous characteristics respectively. Prevalence and its 95% confidence intervals (95%CI) of LSM ≥ 8 kPa will be computed overall (primary endpoint) and in the subgroup of patients with risk factors. Analogously, prevalence of CAP ≥ 250 dB/m and its 95%CI will be computed, overall and among patients with risk factors. Concordance between variables (LSM, fibrosis scores, CAP, steatosis scores, liver biopsy) will be computed. Scatter plots and Pearson’ r linear correlation coefficients will be used to explore the relationship between continuous variables, including LSM and fibrosis scores values. Receiver operating characteristic (ROC) curves, using LSM ≥ 8 kPa as the gold standard for fibrosis, will be computed for the different continuous fibrosis scores. Sensitivity, specificity, predictive values and likelihood ratios will be performed using LSM ≥ 8 kPa as the gold standard for fibrosis for different fibrosis categorized scores. Comparison of values obtained with M and XL probes in the assessment of LSM and CAP will be performed both, using the continuous forms of the variables with t tests (or non-parametric tests in the case these variables violate the necessary assumptions), and using the categorical variables using the 8 kPa and 250 dB/m^2^ cut-offs with Chi squared test. In addition, multivariate linear regression models and multivariate logistic regression models will be computed with LSM (or CAP) as the dependent variable (continuous for linear model and categorical for logistic mode) to assess the effect of the M or XL and potential interaction with the probe size. The relative frequency of adverse events to the study procedures will be reported in percent.

### Health economic evaluation

A health economic evaluation of the study will be performed to assess the cost-effectiveness and budget impact of the screening approach. Two models will be performed to assess the cost-effectiveness and budget impact of such an intervention, both of which will be carried out according to the Consolidated Health Economics Evaluation Reporting Standards 2022 (CHEERS) [25].

The first one will be an empirical Markov-chain model, where included population will be classified in each node of the model (depending on the endpoint), and transition probabilities will be obtained from validated liver-disease progression model. Probabilistic sensitivity analysis will be carried out to assess the inherent uncertainty of the model. For the budget impact model, costing based on resource utilization will be modeled to evaluate the viability of such a screening intervention onto national health services. The results of the model will be much more context-specific as they will depend upon NHS silos. The goal of the health economic evaluation is to help in the design of a cost-effective screening intervention that minimizes its budget impact to healthcare providers.

During follow-up at 5 and 10 years for clinical events, the Markov-chain model will be updated, and transition probabilities will be estimated from the 5-year follow-up. Due to the multicentre nature of the study, rather than collecting cost data, comparable resource utilization across hospitals will be used as the unit of account, such as number of visits to the emergency room, admissions and length of stay. The model will be later calibrated and adjusted for each of the participant countries, as incidence, prevalence and costs may vary depending on country characteristics. The comparative effectiveness endpoints to be modelled will include: self-reported EuroQoL 5d-3L, liver progression and mortality.

### Risks and disadvantages for patients

As in any screening study, the principal individual risk for participants will be the potential overdiagnosis of a chronic disease, which may lead to anxiety and unnecessary investigations and hospital visits. The exhaustive evaluation at the second study visit will assess for the presence or absence of chronic liver disease, and therefore reduce this risk of overdiagnosis. Another potential risk is that associated to liver biopsy. Liver biopsy will be performed as a standard of care work-up in patients with chronic liver disease suspected as a result of the imaging, blood tests and/or VCTE evaluation. Liver biopsy is considered a safe procedure when used for diagnosing purposes of non-malignant liver disorders. Adverse events associated with liver biopsy are pain at the puncture site, intraabdominal bleeding, perforation of the gallbladder or bile peritonitis [26]. The most frequent of these complications is intraabdominal bleeding, which occurs in 2.2 out of 1,000 biopsies. In the current study, all liver biopsies will be performed by experienced medical staff at each participating university hospital. There is no expected risk or discomfort related to VCTE or abdominal ultrasonography.

### Ethics

Currently there are no known effective drugs to treat liver fibrosis. However, treatment of the etiological factors of chronic liver diseases can induce fibrosis regression [6]. Detection of liver fibrosis will lead to implementation of effective treatment in chronic viral hepatitis (B and C) and referral to withdrawal programs for alcohol-related liver disease. Lifestyle modification interventions can be implemented in patients with chronic liver disease due to alcohol-related liver disease and NAFLD, the most frequent etiologies in Europe. Patients with risk factors for chronic liver disease but without fibrosis will also be counseled to change detrimental lifestyle habits in order to avoid disease development in the future. We believe that the benefits of participating in this observational clinical study outweigh the risks mentioned in previous sections, and we believe it to be ethically safe. Informed consent is obtained from all participants.

## Discussion

The present study, an investigator-initiated study held by the LiverScreen consortium and funded by the European Commission H2020 program, aims to investigate the use of a screening program with a non-invasive test for liver fibrosis in the European general population. This study comes at an especially important time, as the burden of chronic liver diseases is expected to increase in coming years [2]. There is thus an urgent need to change the current approach, from diagnosing liver diseases late, when the impact of interventions is limited, to diagnosing the disease earlier, when complications have not yet occurred [2, 14]. This change would facilitate the application of specific therapies that may halt the progression of fibrosis in some patients and prevent them from reaching the stage of decompensated cirrhosis or developing liver cancer. The current study will specifically aim to answer questions regarding diagnostic accuracy and cost-effectiveness of non-invasive tests in the diagnosis of liver fibrosis in a low prevalence setting. Ultimately, the LiverScreen study will serve as a basis from which diagnostic pathways can be developed and adapted to each country.

### Study Status

The study started enrolling participants in May 2018. As of March 2022, 14,539 subjects have been enrolled. The last subjects are expected to be enrolled in June 2023. Follow-up of patients in the longitudinal study will end 10 years after visit 1, up until June 2033.

## Supplementary Information

**Additional file 1. **LiverScreen Project recruiting centers.

**Additional file 2. **Safety assessment, Data collection and processing, Quality control and regulatory considerations.

**Additional file 3. **Participant information sheet.

## Data Availability

Not applicable. For more details regarding Safety assessment, Data collection and processing, Quality control and Regulatory considerations see Additional File 2.

## Abbreviations

APRI: AST to platelet ratio index
CAP: Controlled attenuation parameter
DALY: Disability-associated life years
FIB-4: Fibrosis-4
FLI: Fatty liver index
HSI: Hepatic steatosis index
ION: Index of NASH
LAP: Lipid accumulation product
LSM: Liver stiffness measurement
NAFLD: Non-alcoholic fatty liver disease
NAFLD-LFS: NAFLD-liver fat score
NFS: NAFLD fibrosis score
NASH: Non-alcoholic steatohepatitis
VCTE: Vibration-controlled transient elastography

## References

[1] Abbafati C, Abbas KM, Abbasi-Kangevari M, Abd-Allah F, Abdelalim A, Abdollahi M (2020). Global burden of 369 diseases and injuries in 204 countries and territories, 1990-2019: a systematic analysis for the Global Burden of Disease Study 2019. Lancet (London, England).

[2] Karlsen TH, Sheron N, Zelber-Sagi S, Carrieri P, Dusheiko G, Bugianesi E (2022). The EASL–Lancet Liver Commission: protecting the next generation of Europeans against liver disease complications and premature mortality. Lancet.

[3] Ginès P, Krag A, Abraldes JG, Solà E, Fabrellas N, Kamath PS (2021). Liver cirrhosis. Lancet (London, England).

[4] Angulo P, Kleiner DE, Dam-Larsen S, Adams LA, Bjornsson ES, Charatcharoenwitthaya P (2015). Liver fibrosis, but no other histologic features, is associated with long-term outcomes of patients with nonalcoholic fatty liver disease. Gastroenterology.

[5] Hagström H, Nasr P, Ekstedt M, Hammar U, Stål P, Hultcrantz R (2017). Fibrosis stage but not NASH predicts mortality and time to development of severe liver disease in biopsy-proven NAFLD. J Hepatol.

[6] Schuppan D, Surabattula R, Wang XY (2018). Determinants of fibrosis progression and regression in NASH. J Hepatol.

[7] Singh S, Muir AJ, Dieterich DT, Falck-Ytter YT (2017). American gastroenterological association institute technical review on the role of elastography in chronic liver diseases. Gastroenterology.

[8] Castera L, Yuen Chan HL, Arrese M, Afdhal N, Bedossa P, Friedrich-Rust M (2015). EASL-ALEH clinical practice guidelines: non-invasive tests for evaluation of liver disease severity and prognosis. J Hepatol.

[9] Roulot D, Costes JL, Buyck JF, Warzocha U, Gambier N, Czernichow S (2011). Transient elastography as a screening tool for liver fibrosis and cirrhosis in a community-based population aged over 45 years. Gut.

[10] Berzigotti A, Tsochatzis E, Boursier J, Castera L, Cazzagon N, Friedrich-Rust M (2021). EASL Clinical Practice Guidelines on non-invasive tests for evaluation of liver disease severity and prognosis – 2021 update. J Hepatol.

[11] Fabrellas N, Alemany M, Urquizu M, Bartres C, Pera G, Juvé E (2013). Using transient elastography to detect chronic liver diseases in a primary care nurse consultancy. Nurs Res.

[12] Serra-Burriel M, Graupera I, Torán P, Thiele M, Roulot D, Wai-Sun Wong V (2019). Transient elastography for screening of liver fibrosis: Cost-effectiveness analysis from six prospective cohorts in Europe and Asia. J Hepatol.

[13] Marchesini G, Day CP, Dufour JF, Canbay A, Nobili V, Ratziu V (2016). EASL-EASD-EASO Clinical Practice Guidelines for the management of non-alcoholic fatty liver disease. J Hepatol.

[14] Ginès P, Castera L, Lammert F, Graupera I, Serra-Burriel M, Allen AM (2022). Population screening for liver fibrosis: toward early diagnosis and intervention for chronic liver diseases. Hepatology.

[15] Ginès P, Graupera I, Lammert F, Angeli P, Caballeria L, Krag A (2016). Screening for liver fibrosis in the general population: a call for action. Lancet Gastroenterol Hepatol.

[16] Castéra L, Vergniol J, Foucher J, le Bail B, Chanteloup E, Haaser M (2005). Prospective comparison of transient elastography, Fibrotest, APRI, and liver biopsy for the assessment of fibrosis in chronic hepatitis C. Gastroenterology.

[17] Mathurin P, Hadengue A, Bataller R, Addolorato G, Burra P, Burt A (2012). EASL clinical practical guidelines: management of alcoholic liver disease. J Hepatol.

[18] Lampertico P, Agarwal K, Berg T, Buti M, Janssen HLA, Papatheodoridis G (2017). EASL 2017 clinical practice guidelines on the management of hepatitis B virus infection. J Hepatol.

[19] Mutimer D, Aghemo A, Diepolder H, Negro F, Robaeys G, Ryder S (2014). EASL clinical practice guidelines: management of hepatitis C virus infection. J Hepatol.

[20] Lohse AW, Chazouillères O, Dalekos G, Drenth J, Heneghan M, Hofer H (2015). EASL clinical practice guidelines: autoimmune hepatitis. J Hepatol.

[21] Bedossa P, Poitou C, Veyrie N, Bouillot JL, Basdevant A, Paradis V (2012). Histopathological algorithm and scoring system for evaluation of liver lesions in morbidly obese patients. Hepatology.

[22] Karlas T, Petroff D, Sasso M, Fan JG, Mi YQ, de Lédinghen V (2017). Individual patient data meta-analysis of controlled attenuation parameter (CAP) technology for assessing steatosis. J Hepatol.

[23] Saadeh S, Younossi ZM, Remer EM, Gramlich T, Ong JP, Hurley M (2002). The utility of radiological imaging in nonalcoholic fatty liver disease. Gastroenterology.

[24] Stern C, Castera L (2017). Non-invasive diagnosis of hepatic steatosis. Hepatol Int.

[25] Husereau D, Drummond M, Augustovski F, de Bekker-Grob E, Briggs AH, Carswell C (2022). Consolidated health economic evaluation reporting standards 2022 (CHEERS 2022) statement: updated reporting guidance for health economic evaluations. BMC Health Serv Res.

[26] West J, Card TR (2010). Reduced mortality rates following elective percutaneous liver biopsies. Gastroenterology.

