# Correction: LiverScreen project: study protocol for screening for liver fibrosis in the general population in European countries

**DOI:** 10.1186/s12889-023-15867-6

**Published:** 2023-05-22

**Authors:** Isabel Graupera, Maja Thiele, Ann T. Ma, Miquel Serra-Burriel, Judit Pich, Núria Fabrellas, Llorenç Caballeria, Robert J. de Knegt, Ivica Grgurevic, Mathias Reichert, Dominique Roulot, Jörn M. Schattenberg, Juan M. Pericas, Paolo Angeli, Emmanuel A. Tsochatzis, Indra Neil Guha, Montserrat Garcia-Retortillo, Rosa M. Morillas, Rosario Hernández, Jordi Hoyo, Matilde Fuentes, Anita Madir, Adrià Juanola, Anna Soria, Marta Juan, Marta Carol, Alba Diaz, Sönke Detlefsen, Pere Toran, Guillem Pera, Céline Fournier, Anne Llorca, Phillip N. Newsome, Michael Manns, Harry J. de Koning, Feliu Serra-Burriel, Fernando Cucchietti, Anita Arslanow, Marko Korenjak, Laurens van Kleef, Josep Lluis Falcó, Patrick S. Kamath, Tom H. Karlsen, Laurent Castera, Frank Lammert, Aleksander Krag, Pere Ginès, for the LiverScreen Consortium investigators

**Affiliations:** 1 Liver Unit Hospital Clínic, University of Barcelona, Barcelona, Catalonia Spain; 2 Institut D’Investigacions Biomèdiques August Pi I Sunyer (IDIBAPS), Barcelona, Spain; 3 Centro de Investigación En Red de Enfermedades Hepáticas Y Digestivas (Ciberehd), Barcelona, Spain; 4 Faculty of Medicine and Health Sciences, University of Barcelona, Barcelona, Spain; 5 Centre for Liver Research, Department of Gastroenterology and Hepatology, Odense University Hospital, and Institute for Clinical Research, University of Southern Denmark Odense, Odense, Denmark; 6 Epidemiology, Statistics, and Prevention Institute, University of Zurich, Zurich, Switzerland; 7 Clinical Trial Unit, Hospital Clínic, 08036 Barcelona, Spain; 8 Unitat de Suport a La Recerca Metropolitana Nord, Fundació Institut Universitari Per a La Recerca a L’Atenció Primària de Salut Jordi Gol I Gurina (IDIAPJGol), Metropolitana Nord, IDIAP Jordi Gol, ICS Institut Català de La Salut, Barcelona, Spain; 9 Department of Gastroenterology and Hepatology, Erasmus MC University Medical Centre, Rotterdam, the Netherlands; 10 Department of Gastroenterology, Hepatology and Clinical Nutrition, University Hospital Dubrava, University of Zagreb School of Medicine and Faculty of Pharmacy and Biochemistry, Zagreb, Croatia; 11 Department of Medicine II, Saarland University Medical Center, Homburg, Germany; 12 Unité d’Hépatologie, Hôpital Avicenne, AP-HP, Université Paris 13, Bobigny, France; 13 Metabolic Liver Research Program, Department of Internal Medicine I, University Medical Centre of the Johannes Gutenberg, Mainz, Germany; 14 Liver Unit, Department of Internal Medicine, Hospital Universitari Vall d´Hebron, Vall d’Hebron Institut de Recerca (VHIR), Vall d’Hebron Barcelona Hospital Campus, Barcelona, Spain; 15 Universitat Autònoma de Barcelona, Barcelona, Spain; 16 Unit of Internal Medicine and Hepatology (UIMH), Department of Medicine (DIMED), University-Teaching Hospital of Padova, Padua, Italy; 17 UCL Institute for Liver and Digestive Health, Royal Free Hospital, University College of London (UCL), London, UK; 18 NIHR Nottingham Biomedical Research University Mainz Centre, Nottingham University Hospitals NHS Trust and the University of Nottingham, Nottingham, UK; 19 Liver Section, Gastroenterology Department, Hospital del Mar, Department of Medicine, IMIM, Barcelona, Spain; 20 Liver Unit, Hospital Germans Trias I Pujol, IGTP, Badalona, Spain; 21 Institut Catala de La Salut (ICS), BCN. Ambit d’Atencio Primaria, Barcelona, Spain; 22 Department of Pathology, Centre of Biomedical Diagnosis, Hospital Cínic, Barcelona, Spain; 23 Department of Pathology, Odense University Hospital (OUH), University of Southern Denmark, Odense, Denmark; 24 Echosens, Paris, France; 25 National Institute for Health Research Biomedical Research Centre at University Hospitals Birmingham NHS Foundation Trust and the University of Birmingham, Birmingham, UK; 26 Health Sciences, Hannover Medical School MHH, Hannover, Germany; 27 Department of Public Health, Erasmus University Medical Center, Rotterdam, the Netherlands; 28 Barcelona Super Computing Center (BSC), Barcelona, Spain; 29 European Liver Patients’ Association, Brussels, Belgium; 30 Genesis Biomed, Barcelona, Spain; 31 Division of Gastroenterology and Hepatology, Mayo Clinic College of Medicine and Science, Rochester, MN USA; 32 Oslo University Hospital, Oslo, Norway; 33 Department of Hepatology, Hôpital Beaujon, Assistance Publique-Hôpitaux de Paris, Clichy, Université de Paris, Paris, France; 34 Institute for Occupational Medicine and Public Health, Saarland University, Homburg, Germany; 35 Hannover Medical School (MHH), Hannover, Germany

**Correction: BMC Public Health 22, 1385 (2022)**

**https://doi.org/10.1186/s12889-022-13724-6**

The original publication of this article [1] contained an incomplete author contribution section. The incorrect and correct information has been listed in this correction article. The original article has been updated.

Incorrect

IG, MT, MS, JP, NF, LC, FL, AK, NG, ET, PG conceptualised the project. All authors were involved with generation of the protocol. IG, MT, ATM, MS, GP and JP wrote the first draft of the manuscript. All authors listed have been implicated in the development of the ongoing project described in the protocol including patients. All authors were involved in editing and approving the manuscript. The corresponding author attests that all listed authors meet authorship criteria and that no others meeting the criteria have been omitted. IG, MT, MS, and AK act as guarantors.

Correct.

IG, MT, MS, JP, NF, GP,LC, FL, AK, NG, ET, PG conceptualised the project. All authors were involved with generation of the protocol. IG, MT, ATM, MS, GP and JP wrote the first draft of the manuscript. All authors listed (IG,MT,AM,MS,JP,NF, LC,RK,IG,MR,DR,JS,JP,PA,ET,IG,MG,RM,RH,JH,MF,AM,AJ,AS,MJ,MC,AD,SD,PT,CF,AL,PN,MM,HK,FS,FC,AA,MK,LK,JF,PK,TK,LC,FL,AK,PG) have been implicated in the development of the ongoing project described in the protocol including patients. All authors(IG,MT,AM,MS,JP,NF,LC,RK,IG,MR,DR,JS,JP,PA,ET,IG,MG,RM,RH,JH,MF,AM,AJ,AS,MJ,MC,AD,SD,PT,CF,AL,PN,MM,HK,FS,FC,AA,MK,LK,JF,PK,TK,LC,FL,AK,PG) were involved in editing and approving the manuscript. The corresponding author attests that all listed authors meet authorship criteria and that no others meeting the criteria have been omitted. IG, MT, MS, and AK act as guarantors.
