# Supplementary material for: LiverScreen project: study protocol for screening for liver fibrosis in the general population in European countries
Source: BMC Public Health. 2022 Jul 19;22:1385. doi: 10.1186/s12889-022-13724-6 (PMC9295430; doi:10.1186/s12889-022-13724-6)
Supplement: Supplementary file 1 — Additional file 1. LiverScreen Project recruiting centers. [file 12889_2022_13724_MOESM1_ESM.docx]

**Additional File 1** LiverScreen Project recruiting centers

| **Country** | **Reference center** | **Primary Care recruitment centre** |
| --- | --- | --- |
| Croatia | **University of Zagreb School of Medicine (UZSM)**   - Hospital Dubrava | Poliklinika Dubrava |
| Denmark | **Odense Universitetshospital (OUH)** | **Odense Universitetshospital (OUH)** |
| France | **Assistance Publique – Hôpitaux de Paris (AP-HP)**   - Hôpital Avicenne - Hôpital Beaujon | CRC Avicenne  Gennevilliers |
| Germany | **Universitaetsmedizin der Johannes Gutenberg-Universitaet Mainz** **(UMCU)** | Mainz Praxis Mombach  Mainz Altstadtpraxis  Mainz Praxis Schneider  Mainz University Hospital |
| Germany | [**Universität des Saarlandes**](https://www.uni-saarland.de/start.html) (**USAAR**) | University Medical Centre Homburg (UKS) Praxis Lißmann Homburg  Praxis Kaiser St. Ingbert |
| Italy | **Azienda Ospedaliera di Padova** **(AOP)**   - University of Padua | Vigonza Medica  Medicina di Gruppo di Montagnana |
| Netherlands | Erasmus MC University Medical Center, Rotterdam (EMC) | Erasmus MC University Medical Center, Rotterdam (EMC) |
| Spain | **Fundació Clínic per a la Recerca Biomèdica (FCRB)**   - **Hospital Clínic Barcelona** | CAP La Marina  CAP Numància  CAP Bordeta/Magòria  CAP Adrià  SEAT |
| Spain | **Institut Català de la Salut (ICS)**   - Hospital Germans Trias i Pujol - IDIAP Jordi Gol - Institut de Recerca Vall d’Hebron (VHIR) | CAP II Maresme-Mataró  CAP La Florida  CAP II Santa Coloma  CAP La Llàntia  CAP Sant Rafael  CAP Río de Janeiro  CAP Ciutat Meridiana |
| Spain | **Consorci Mar Parc de Salut de Barcelona (IMIM**)   - Hospital del Mar | CAP Vila Olímpica  CAP Barceloneta  CAP Besòs |
| UK | **The University of Nottingham (NIHR**) | King’s Mill Hospital |
| UK | **University College London** **(UCL)**   - Royal Free Hospital | Royal Free Hospital |
