# Supplementary material for: LiverScreen project: study protocol for screening for liver fibrosis in the general population in European countries
Source: BMC Public Health. 2022 Jul 19;22:1385. doi: 10.1186/s12889-022-13724-6 (PMC9295430; doi:10.1186/s12889-022-13724-6)
Supplement: Supplementary file 2 — Additional file 2. Safety assessment, Data collection and processing, Quality control and regulatory considerations. [file 12889_2022_13724_MOESM2_ESM.docx]

# **Addition File 2. Safety assessment, Data collection and processing, Quality control and regulatory considerations.**

# **Safety assessment:**

The purpose of this study is not to investigate any pharmaceutical or investigational product. Only TE and liver biopsy related adverse events (RAE) and serious adverse events (SAE) that occur within the first month after each study visit will be recorded, based on careful clinical observation of the patient, laboratory tests or spontaneously reports by the participant discovered as a result of general questioning by the study staff.

TE and liver biopsy RAE and SAEs will be recorded on the medical history and in the CRF.

Follow-up of the TE and liver biopsy RAE and SAE is required until the event resolves or stabilizes at a level acceptable to the Investigator. The liver biopsy RAE and SAEs that have not resolved by the end of the study, or that have not resolved upon discontinuation of the subject's participation in the study, will be followed until any of the following occurs: the event resolves, the event stabilizes, the event returns to baseline, if a baseline value/status is available, the event can be attributed to other factors than the study procedure or to factors unrelated to study conduct, it becomes unlikely that any additional information can be obtained (subject or health care practitioner refusal to provide additional information, lost to follow-up after demonstration of due diligence with follow-up efforts).

The principal investigator or delegates of each center will report immediately any SAE occurring from signing of informed consent and up to 30 days after each study visit to the Coordinating Center.

As required by local regulations, the principal investigator will also notify immediately all SAEs to the Local Ethics Committee.

SAEs forms will be sent immediately by fax or email to: CTU CLINIC at Hospital Clinic de Barcelona.

# **Data collection and processing**

## Recording of data

All data required for the study will be recorded in participating centers using a web-based electronic CRF (eCRF) collection tool. Completeness and plausibility checks will ensure the collection of high quality data.

An e-CRF for each patient will be completed by authorized personnel who must be identified and authorized in writing by the Principal Investigator before they conduct any study related tasks. A delegation of responsibility log identifying who can enter data and/or sign off a CRF will be maintained by the Principal Investigator.

The subject’s number and date of entry into the study, along with a study identifier, should be recorded in the subject’s study records. The following should also be recorded in the study records; confirmation of written consent, the subject’s clinical status, date of every study visit, copies of all relevant reports and laboratory tests, comments on results and reference to serious adverse events and related adverse events.

Direct access to source data/documents

Investigators will ensure access to the source documents of the staff responsible for guaranteeing data quality and data analysis. In addition, access to documentation will be provided, if necessary, to the staff duly authorized by the sponsor (study monitors).

## Data management

Each Investigator will ensure the accuracy, completeness, legibility and timelines of data reported in the CRF and all required reports. Any change or correction to the CRF must be dates, initialed and explained (if necessary).

## Archiving and storage of data

Each investigator is responsible for maintaining all records which enable the conduct of the study at the site to be fully documented, in accordance applicable national regulatory requirements. Timeliness and completeness of the documentation will be regularly checked by the clinical monitor. All completed study related documents (e.g. eCRF, Informed consent forms, Subject identification log, etc) must be archived at site.

# **Study quality control**

A monitoring plan has been designed to establish the guideline for conducting all the monitoring activities. The CTU (Clinical Trials Unit) in Hospital Clinic will perform the monitoring and will report directly to the Steering Committee of the LiverScreen consortium. No specific data monitoring committee (DMC) has been created for the project. The report of the monitoring of the data by the CTU will be evaluated by the LiverScreen Steering Committee.

Briefly, source data will be verified during on-site or/and on-line monitoring visits. During the visits, the monitor will compare the data entered into the eCRF with the source documents. Because the sample size is composed of 30.000 subjects, only variables related to the primary and secondary outcomes will be monitored on-site in all patients. All remaining variables will be monitored on-line. The nature and location of all source documents will be identified to ensure that all sources of original data required to complete the eCRF are known to the monitor and study-site personnel and are accessible for verification. During monitoring visits, the relevant study-site personnel should be available, the source documentation accessible, and a suitable environment provided for review of sub-related documents. The monitor will meet the investigator. The number of visits and nature of visits (remote visits) will be described in detail in the monitoring plan. A close-out visit will be performed before the final closure of each center. Besides, a general overview and a final revision of pending queries will be implemented, ensuring that all data and corresponding source documents are in place before the site closure.

#

# **Regulatory considerations**

### Responsibilities of the investigators

Each investigator is responsible for ensuring that the study is performed in accordance with the protocol and applicable regulatory and country-specific requirements. In addition, the investigator is responsible for giving information about the study to all staff members involved in the study or in any element of subject management, before and during the study. The investigator is also responsible for ensuring the privacy, health, and welfare of the subjects during and after the study.

### Informed consent

Each subject included will give written consent according to local requirements after the nature of the study has been fully explained. The ICF(s) will be signed before performance of any study related activity. Each country has its own ICFs approved by the reviewing Ethics Committee and is in a language that the patient can read and understand. The informed consent is in accordance with principles that originated in the Declaration of Helsinki (updated according to its last version, Fortaleza, Brazil, 2013), applicable regulatory and country-specific requirements. By signing the ICF the subject is authorizing such access, including permission to obtain information about his or her survival status, and agrees to allow his or her study physician to re-contact the subject to obtain consent for additional safety evaluations, if needed, and subsequent disease-related treatments, or to obtain information about his or her survival status.

## Patient confidentiality

The collection and processing of personal data from subjects enrolled in this study will be limited to those data that are necessary to fulfill the objectives of the study. Patient data will be codified to ensure confidentiality and compliance with applicable data privacy protection laws and regulations. Subjects will be codified with a study code that prevents their identity from being deduced. The principal investigator of each center and duly authorized collaborators will compromise to maintain personal data strictly confidential, according to the corresponding country-specific requirements. In the case report form, the patient will only be identified by the assigned study code. The name of patients will not appear in any publication or report of the study results. The participation of the patient in the study will be noted in their medical records. The investigator will complete a list which will include the names of the patients participating in the study, the number of inclusion in the study, and their medical history. Only investigators and the staff responsible for guaranteeing data quality and data analysis will have access to the clinical documentation of the participants. Duly authorized persons by the sponsor and the health authorities and the Ethics Committee may audit or inspect the study. Personal information will not be publicly available, in compliance with Personal Data Protection regulation. The subject has the right to request through the investigator access to his or her personal data and the right to request rectification of any data that are not correct or complete pursuant to European requirements (EU Directive 96/46 on data protection). According to the above law, patients can exercise their rights to data access, rectification, opposition, and cancellation, for which they must contact the study doctor. Privacy and confidentiality of data generated in the future on stored samples will be protected by the same standards applicable to all other clinical data. Only data collected for the study that does not bear any information that could directly identify the patient will be transferred to third parties or other countries. Should this transfer occur, it will be for the same purposes as the study and guarantee confidentiality with at least the level of protection afforded by applicable regulations in Spain. Patients will be informed that their clinical data will be incorporated into an automated study specific file after and the results of the study and different sub-studies conducted with samples can be communicated at scientific meetings, medical conferences or publications. However, patient’s identity or identifiable data will never be disclosed.
