# Supplementary material for: LiverScreen project: study protocol for screening for liver fibrosis in the general population in European countries
Source: BMC Public Health. 2022 Jul 19;22:1385. doi: 10.1186/s12889-022-13724-6 (PMC9295430; doi:10.1186/s12889-022-13724-6)
Supplement: Supplementary file 3 — Additional file 3. Participant information sheet. [file 12889_2022_13724_MOESM3_ESM.pdf]

PARTICIPANT INFORMATION SHEET

Study title: Screening for liver fibrosis. A population-based study in European countries. The ''LiverScreen'' project.

Working title: Screening for liver fibrosis

Protocol code: LiverScreen

Sponsor: Fundació Clínic per a la Recerca Biomèdica (FCRB), Barcelona, Spain.

Principal investigator: ……………………………………………………………………………

Site: ……………………………………………………………………………………………………

Invitation

We invite you to participate in a clinical research study called the LIVERSCREEN project study, in which we are evaluating the usefulness and feasibility of a screening program to detect liver fibrosis by liver stiffness measurement among European general population.

Before deciding whether you want to take part in this study, it is important for you to understand why this study is being done. Please read this information carefully and discuss it with friends, relatives and your doctor if you consider necessary. Please ask us if there is anything that is not clear or if you require further information.

What is the purpose of this study?

The main purpose of this study is to assess the prevalence of liver fibrosis in general population using FibroScan®, a simple and widely available non-invasive method that measures liver stiffness measurement and identify liver fibrosis.

Liver cirrhosis is the end-stage of all causes of chronic liver diseases and is associated with high morbidity and mortality. Chronic liver injury cause inflammation and fibrosis and the accumulation of fibrosis leads to cirrhosis over a period of 2-3 decades. In general, patients are not diagnosed during this period of time because the disease is asymptomatic, and patients do not seek medical attention. The main factor predicting long-term outcome of patients with chronic liver disease in early stages is the existence of liver fibrosis. There are no strategies for early detection of cirrhosis before decompensation or cancer occurs. This is important because therapies are less effective in late stages compared to early stages.

Standard liver tests used to evaluate liver function are not accurate methods to detect fibrosis. In recent years, a new non-invasive method assessing the presence and severity of liver fibrosis has been developed, FibroScan®. This method relies on liver stiffness measurement. FibroScan® is a widely available point-of-care technique that can be performed in 5-10 minutes, with no pain and no sedation. This technique thus seems particularly suited for the early detection of chronic liver diseases.

We have designed this clinical research study to investigate if FibroScan® is useful as a screening method for liver fibrosis detection in general population.

Why have I been invited?

You have been invited to take part in this study because this is a population-based study and we have selected randomly subjects from general population or because your reference nurse

or general practitioner have invited you. You have been invited to participate in this study at random, not because you have any risk factors for chronic liver disease.

Do I have to take part?

No. Your participation in this study is entirely voluntary. If you decide to take part, you are free to withdraw from the study at any time without giving a reason. Importantly, a decision to withdraw from the study or a decision not to take part will not affect the care that you receive ordinarily and regularly from your doctors and nurses. Please note that data and samples obtained during the period of your involvement in the study will be stored and used for analysis unless you ask for it to be destroyed. However, it might not be possible to return your samples or remove your data from the study results if they have already been processed at the time you decide to withdraw from the study.

What will happen to me if I take part?

If you decide to participate, you will be asked to sign the Consent Form attached at the end of this document.

A total of 20.000 participants from different sites in Spain, France, Germany, Italy, The Netherlands, Denmark and UK will take part in this study over a 2 year period. Your participation will take between 1 to 3 months and you will have to go to your primary care centre and your reference hospital 1 to 3 times depending on the result of the first visit.

If you decide to participate and you are eligible for this study you will undergo a first visit in which a nurse will discuss your medical history with you, recording any medications you are currently taking, and performing a physical examination including vital signs. During this visit we will ask you to complete three questionnaires related to quality of life, health status and alcohol consumption. Blood tests will also be obtained. The total amount of blood drawn at this visit will be around 16,7mL. A FibroScan® exam will be done. This exam measures liver stiffness and will take 10 minutes. To perform the exam, you will be lying, and a transducer probe will be placed on the skin between two ribs at the level of the right lobe.

If all blood tests and FibroScan® are normal in this first visit, your participation in the study will be over. On the contrary, if the result of the FibroScan® suggests that you may have significant liver fibrosis (≥8Kpa) and/or you have increased liver enzyme tests and/or the FibroScan® fails to obtain valid results you will be subsequently evaluated in a second visit at the University Hospital within the following three months to confirm the diagnosis of liver disease and assess the etiology.

In addition, we will ask you to obtain an extra blood sample of 5 ml for genetic tests. The genetic test will be to perform whole-genome sequential methods in your blood samples aimed at identify genetic variants among individuals associated to liver fibrosis and chronic liver diseases.

The second visit will be performed by hepatologists according to a standardized work-up including complete blood liver tests, FibroScan® and liver ultrasound. A liver biopsy will be proposed to you for diagnosis and staging of liver disease as common practice for liver diseases. Performing a liver biopsy is usual clinical practice when FibroScan® is elevated to confirm the diagnosis of the disease. If you decide not to undergo the biopsy for any reason, your doctor will also monitor the disease clinically.The total amount of blood drawn at this visit will be around 11 mL.

After 5 and 10 years, a member of the investigator team will contact you by phone to evaluate possible changes in your health status, mainly related to liver and cardiovascular events that might have accounted during this period.

What are the possible side effects of any procedure undertaken when participating in the study?

Blood drawing may be associated with discomfort and may leave a temporary bruise. Every effort will be made to minimise this.

FibroScan® consists of an ultrasound transducer mounted on the axis of the vibrator, which produces vibration of a mild amplitude and low frequency (50 Hz), consequently inducing elastic shear wave that propagates through the liver. Pulse-echo ultrasound follows the propagation of the shear wave and measures its velocity, which is related to liver tissue stiffness. The propagation velocity of a wave is correlated with the amount of fibrosis in the liver. Performance of FibroScan® takes only a few minutes, and it is well tolerated by most patients. The full exam requires for the operator to obtain 10 measurements of stiffness (in kPa) from the person by pressing a probe in between the ribs. This technique does not represent any potential risk of side effects.

Abdominal ultrasound is a type of imaging test that uses transmission and reflection of ultrasound waves to visualise internal organs (liver, gallbladder, spleen, etc…) through the abdominal wall (with the help of gel which helps transmission of the sound waves). You will be lying down for the procedure. A clear, water-based conducting gel is applied to the skin over the abdomen. This helps with the transmission of the sound waves. The procedure takes 30 minutes approximately. Ultrasound does not represent any potential risk of side effects.

A percutaneous liver biopsy is a procedure that involves putting a thin needle through the abdomen and into the liver to remove a small piece of liver tissue, so it can be examined under a microscope for signs of damage or disease. Local anesthesia is used to perform liver biopsy. The major risk of liver biopsy is bleeding from the site of needle entry into the liver, although this occurs in less than 1% of patients. Other possible complications include infection, puncture of other organs and damage of the gallbladder, although all these complications are rare because percutaneous liver biopsy is performed under ultrasound vision. The risk of death from liver biopsy is extremely low, with a mortality of 1 in 5.000.

What are the possible benefits of taking part?

There is no guaranteed benefit to you as a result of your participation in this study. However, information gained from this study will help us to improve early detection of chronic liver diseases. On the other hand, if you are diagnosed of chronic liver disease with fibrosis, you will benefit from an early diagnosis and can therefore be offered treatment for your disease.

Expenses and payments

Your participation in the study will not involve any expenditure for you. You will not receive any compensation for your participation in this study.

What if there is a problem?

If you have a concern about any aspect of this study, you should ask to speak to the researchers who will do their best to answer your questions. You will find their contact details in this information sheet.

Will my taking part in the study be kept confidential?

Yes. All information collected about you during the study will be kept strictly confidential. Any study information about you that leaves the primary care or the hospital will have your name and address removed so that you cannot be recognized from it.

We have made sure that everyone involved in the study follow the laws and regulations that protect participant confidentiality. Only authorized representatives connected with the study are allowed access to the names of participants in the study. In addition, representatives of the sponsor, funding and regulatory authorities could also review and access to your medical records to check that the study is being carried out correctly.

All records that identify you will be kept confidential and will not be made publicly available. If the results of the study are published, your identity will remain confidential. If reference to you is made, this will only be done by using code numbers.

According to the applicable privacy law, you have the right of access to data relating to yourself. In addition, you have the right to demand for access to and correction of your personal data. For more information, please consult your study doctor.

The information gathered during this study will be processed electronically. By signing the attached informed consent form, you are authorizing such processing of data.

In case of sending your data collected for the study to third parties and to other countries, the data will not contain information that you can identify. If this occurs, it will be done for the purpose of the study and guaranteeing confidentiality as a minimum level of protection of the laws in force your country.

Some of your data will be sent in encrypted form to Echosens, the French manufacturer of FibroScan®. Echosens will use this data internally to improve the FibroScan® technique for use in detecting liver fibrosis in the general population. This cession will be made guaranteeing the confidentiality of the patient at the level of protection required by current legislation in your country.

What will happen to any samples I give?

Blood samples collected for this study will be stored and analysed in the local laboratories at your site. Once analysed, all the samples will be destroyed in accordance with [specify law, if applicable] unless you consent their use for future studies in a separate and specific consent form.

A sample of your liver biopsy will be sent to Hôpital Beaujon, Clichy, France, where an expert anatomopathologist will analyse it.

Anything that can identify you will be removed and your samples will be labelled only with a study code number. All the samples that have been collected from you will be stored and will be used for the purpose of this study.

What will happen to samples for genetic tests I give?

If you consent, an extra blood sample of 5 ml for genetic tests will be obtained during the visit 1. This sample will be collected at your site and sent to the Liver Unit in Hospital Clínic of Barcelona, Spain for their analysis for genetic studies. The samples will be stored in the Hepatorenal Biobank Collection of Hospital Clínic de Barcelona (samples collection registered in the Biobank of Hospital Clínic-IDIBAPS, Barcelona and in the National Register of Biobanks) until their analysis. Once analyzed, the samples will be destroyed in accordance with local regulations unless you consent their use for future studies in a separate and specific consent form.

Samples will be labeled only with a study code number and any data that can identify you will be removed.

What will happen to the results of the research study?

The study results will be presented at international research conferences and published in research journals. The results will be reported in an internal clinical study report which will be sent to the Ethics Committee and Regulatory Authorities. You will not be identified in this report.

Who is organising and funding the research?

This is a clinical research study that has been written and developed by the LiverScreen consortium, a European consortium of 7 European countries including Spain, France, Germany, Italy, The Netherlands, Denmark and United Kingdom. LiverScreen project has received funding from the European Union's EIT Health program and from private funding. The clinical study is sponsored by the Fundació Clínic per a la Recerca Biomèdica (FCRB), Barcelona, Spain.

Who has reviewed the study?

This study has been reviewed and approved by an independent group of people called Ethics Committee. In addition, the study will be carried out in accordance with the Declaration of Helsinki.

What will happen if I don't want to carry on in the study?

You can withdraw from the study at any time but information collected may still be used. Any stored blood or urine sample that can still be identified as yours will be destroyed if you wish.

CONTACT DETAILS FOR FURTHER INFORMATION
Study doctor: …………………………………………………………………….
Contact Telephone number: …………………………………………………

Thank you for reading this information sheet.

CONSENT FORM

Study title: Screening for liver fibrosis. A population-based study in European countries. The ''LiverScreen'' project.

Working title: Screening for liver fibrosis

Protocol code: LiverScreen

Sponsor: Fundació Clínic per a la Recerca Biomèdica (FCRB), Barcelona, Spain.

Principal investigator: ………………………………………………………………………………

Site: ………………………………………………………………………………………………………1. I confirm that I have read and understand the information sheet (version 1.1 dated 9 January 2018) for the above study. I have had the opportunity to consider the information, ask questions and have had these answered satisfactorily.

2. I understand that my participation is voluntary and that I am free to withdraw at any time without giving any reason, without my medical care or legal rights being affected.

3. I understand that relevant sections of any of my medical notes and data collected during the study may be looked at by responsible individuals from the sponsor, from regulatory authorities or from the funding organisation, where it is relevant to my taking part in this research. I give permission for these individuals to have access to my records.

4. I agree to give an extra 5ml blood sample for genetic testing. I agree for these samples to be sent to Hospital Clínic of Barcelona and to be stored in the Hepatorenal Biobank Collection of the Hospital Clínic in Barcelona until their analysis.
Yes ☐ No ☐

5. If I have a liver biopsy, I agree the sample to be sent to Hôpital Beaujon, Clichy, France for its analysis. Yes ☐ No ☐

6. I wish to exercise my right to be informed of the results obtained from the studies carried out using my samples, including when the findings may have a relevant implication for my health or that of my relatives Yes ☐ No ☐

7. I agree to be contacted in the future by the investigator team for obtaining health-related information Yes ☐ No ☐

8. I agree to take part in the above study.

Name of Participant	Date	Signature
Name of Person taking consent (principal or co-investigator)	Date	Signature

When completed, 1 for participant; 1 to be kept in medical notes
